# Supplementary material for: Identification of specific host cell potassium ion channels that mediate escape of bunyamwera virus from late endosome compartments
Source: J Biol Chem. 2026 Jul 28;302(9):113380. doi: 10.1016/j.jbc.2026.113380 (PMC13524552; doi:10.1016/j.jbc.2026.113380)
Supplement: Supplementary file 1 [file mmc1.pdf]

	Normalised rBUNV-EGFP expression (%)			
Target	siRNA 1	siRNA 2	siRNA 3	Median
KCNC3	22	40	25	**25**
SCN2B	23	26	121	**26**
KCNN3	16	27	82	**27**
SCN9A	29	85	11	**29**
KCNA2	30	29	25	**29**
KCNK1	25	30	103	**30**
KCNA1	59	29	30	**30**
KCNK3	31	26	54	**31**
TRPV3	97	22	34	**34**
KCNJ14	35	36	121	**36**
KCNJ5	24	36	37	**36**
KCNMA1	38	78	37	**38**
GRIK5	10	39	41	**39**
SCN3A	39	14	58	**39**
CLIC4	39	93	6	**39**
KCND2	40	5	67	**40**
KCNA6	39	40	148	**40**
ITPR3	27	40	86	**40**
KCNN2	40	84	30	**40**
ATP6V1B1	33	89	41	**41**
KCNQ3	42	29	51	**42**
SCN4A	42	40	95	**42**
KCNJ15	42	34	83	**42**
GRIN2C	42	20	74	**42**
FXYD1	32	71	43	**43**
GRIN2A	23	64	43	**43**
CNGB1	35	43	97	**43**
VDAC3	50	45	38	**45**
KCNAB2	46	39	132	**46**
KCNJ12	36	46	197	**46**
TRPV6	46	83	39	**46**
FXYD3	47	46	50	**47**
PKD2L1	70	47	41	**47**
KCNJ13	75	47	11	**47**
KCTD5	60	31	47	**47**
ASIC3	42	47	60	**47**
KCNC2	44	48	93	**48**
SCN5A	32	67	49	**49**
PKD2	42	49	85	**49**
TPCN1	49	90	17	**49**
GRID1	30	49	148	**49**
SCN1A	39	54	50	**50**
RYR3	42	50	54	**50**
GABRE	50	104	30	**50**
CACNA2D4	41	50	73	**50**
TRPM1	51	50	41	**50**
Mock	0	0	0	**0**
BUNV + TFR	90	100	100	**100**
SCR	85	83	76	**83**
KCNB2 (-ve CTL)	120	100	105	**105**

Supplementary Figure 1. Identification of human ion channels required during BUNV infection using siRNA screening. Table of siRNA ion channel targets which inhibited rBUNV-eGFP expression by over 50% based on eGFP signal across three unique siRNAs. Each siRNA value represents the mean of four experimental repeats, and the median for each target was calculated. Targets are colour-coded by ion selectivity, and the heatmap shows most inhibitory to rBUNV-eGFP expression (red) to least inhibitory (blue). Values for mock cells, BUNV-infected cells treated with the transfection reagent (TFR) only, BUNV-infected cells treated with scrambled siRNA (SCR) are shown as controls, alongside the results for KCNB2 which was chosen as a negative channel control in later experiments due to its lack of impact on rBUNV-eGFP infection.

- **Potassium (K^+) channels**
- **Chloride (Cl^-) channels**
- **Calcium (Ca^{2+}) channels**
- **Sodium (Na^+) channels**
- **Proton (H^+) channels**
- **Cation ($^+$) channels**
- **Anion ($^-$) channels**

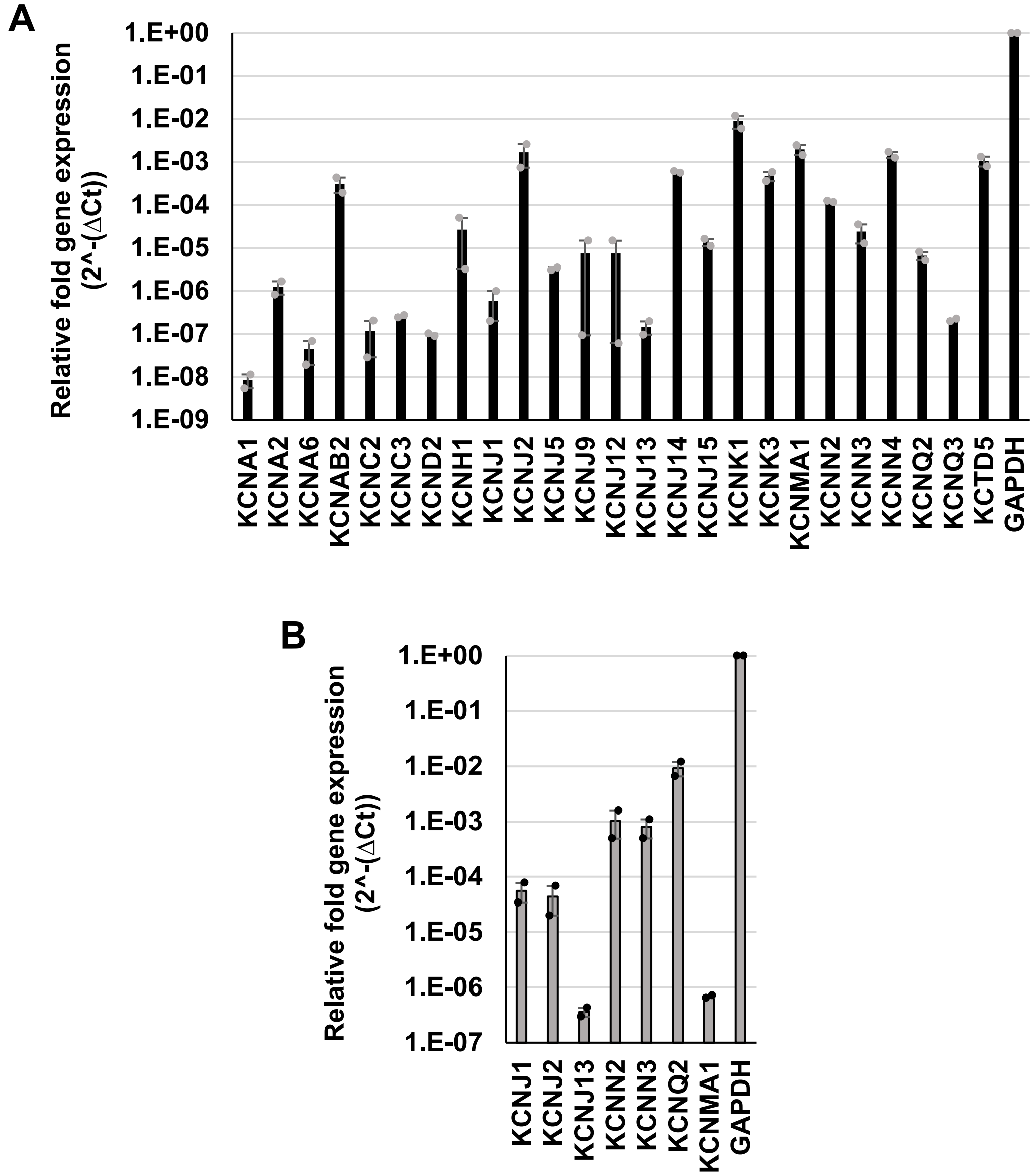

Supplementary Figure 2. Expression of K+ channels in A549 cells. The mRNA expression levels of K+ channel siRNA targets was measured in A549 cells (A) or HEK293-T cells (B) using RT-qPCR. Total RNA was isolated from cells, and gene specific primers were used to quantify mRNA expression relative to the housekeeping gene, GAPDH. Data is a result of 2 experimental and 2 biological repeats.

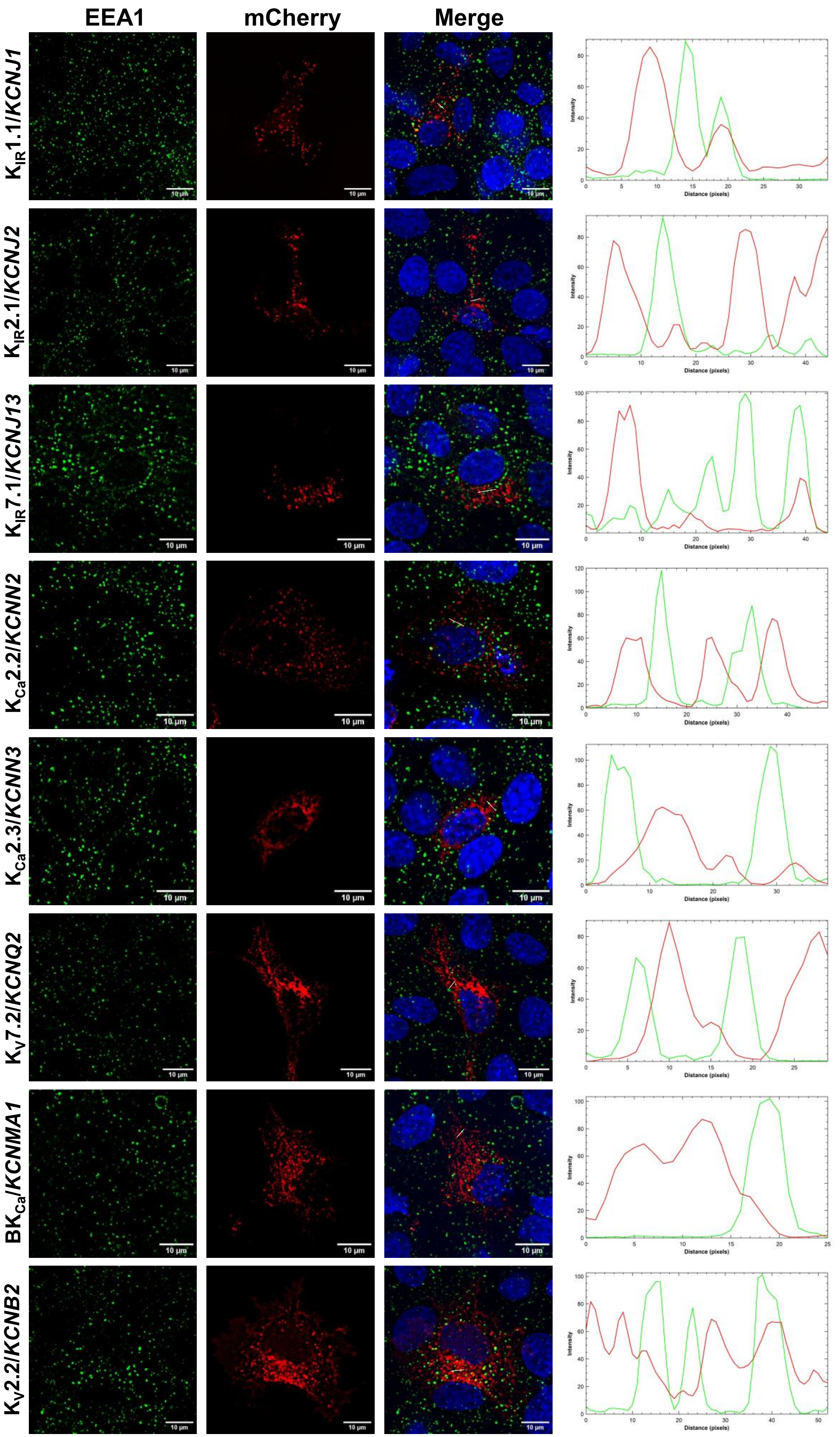
EEA1
mCherry
Merge
$K_{IR}1.1$/*KCNJ1*
$K_{IR}2.1$/*KCNJ2*
$K_{IR}7.1$/*KCNJ13*
$K_{Ca}2.2$/*KCNN2*
$K_{Ca}2.3$/*KCNN3*
$K_{V}7.2$/*KCNQ2*
BK_{Ca}/*KCNMA1*
$K_{V}2.2$/*KCNB2*
10 µm
Intensity
Distance (pixels)

Supplementary Figure 3. K^+ channels do not co-localise with early endosomal marker EEA1. Plasmids encoding WT K^+ channels tagged with mCherry (red) were transfected into A549 cells for 48 hours. Cells were then fixed and immuno-stained for early endosomal marker EEA1 (green). DAPI is also shown (blue). Cells were imaged on an Olympus widefield microscope at 60x magnification. Scale bars (10 μM) were added, and line scan analysis performed, using Fiji software.

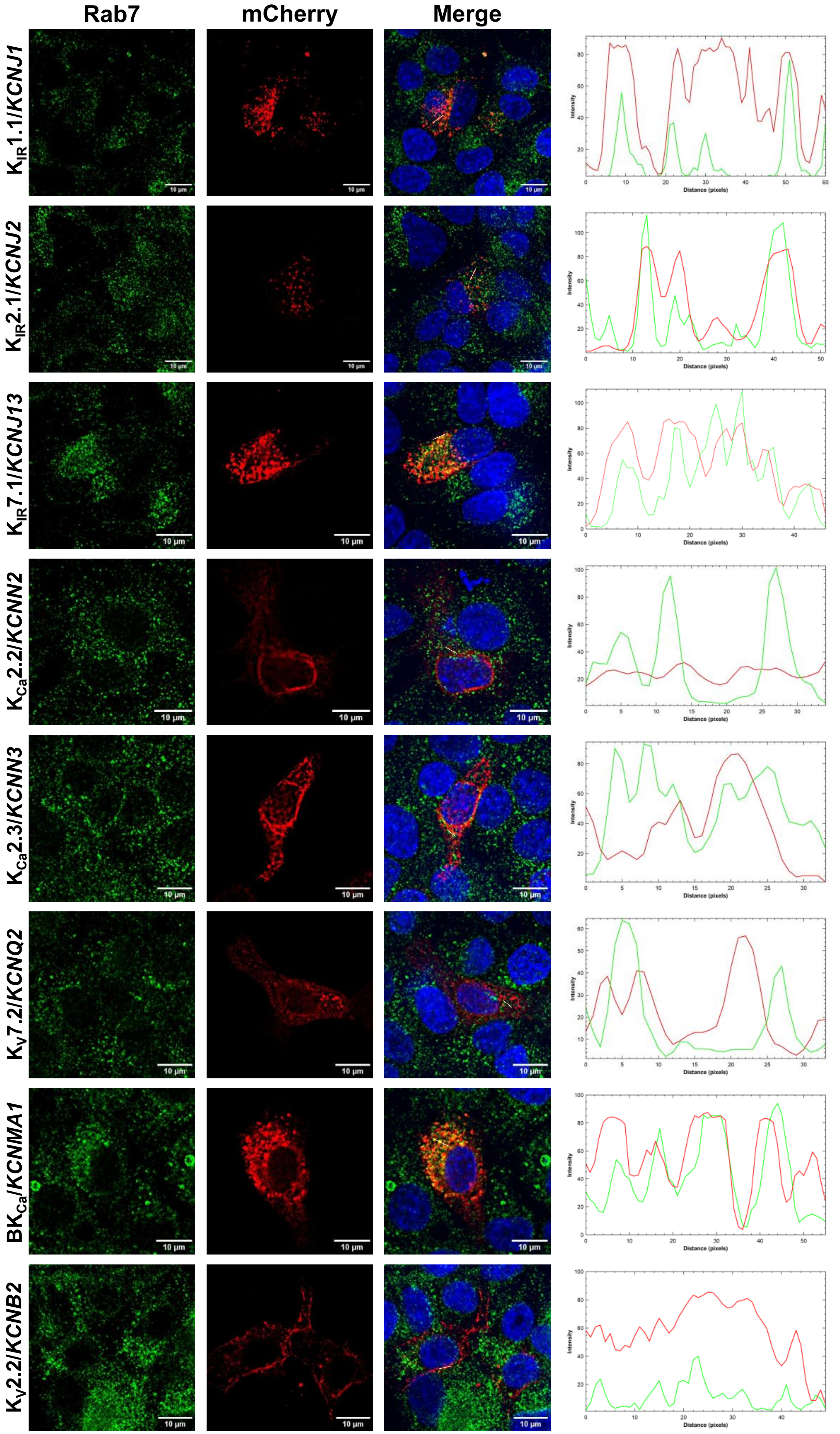

Rab7
mCherry
Merge
K_{IR}1.1/*KCNJ1*
K_{IR}2.1/*KCNJ2*
K_{IR}7.1/*KCNJ13*
K_{Ca}2.2/*KCNN2*
K_{Ca}2.3/*KCNN3*
K_V7.2/*KCNQ2*
BK_{Ca}/*KCNMA1*
K_V2.2/*KCNB2*
10 µm
Intensity
Distance (pixels)

Supplementary Figure 4. Some K^+ channels co-localise with late endosomal marker Rab7. Plasmids encoding WT K^+ channels tagged with mCherry (red) were transfected into A549 cells for 48 hours. Cells were then fixed and immuno-stained for late endosomal marker Rab7 (green). DAPI is also shown (blue). Cells were imaged on Olympus IX83 widefield deconvolution microscope at 60x magnification. Scale bars (10 μM) were added, and line scan analysis performed, using Fiji software.

Target Gene (Protein)	Forward primer (5'-3')	Reverse primer (5'-3')
**KCNA1 (K_V1.1)**	TTACGAGTTGGGCGAGGA	TGACGATGGAGATGAGGATG
**KCNA2 (K_V1.2/BK2)**	ATGAGAGAATTGGGCCTCCT	CCCACTATCTTTCCCCCAAT
**KCNA6 (K_V1.6)**	AGGATGAAGACGATTCCTACACA	AGGGGAAGATAGCCACCAAGT
**KCNAB2 ($K_V$beta-2, shaker-related)**	AGCCAAGCTGAAGGAGCTG	TCATTTCCCGATGACATCAC
**KCNB2 (K_V2.2)**	AACGAAGAACTGAGGCGAGAG	CAAAGATCCTGGCCATCGTG
**KCNC2 (K_V3.2)**	GAAACCAAAGCAGAACCACT	GGTGCCCCCGACATTGAGGA
**KCNC3 (K_V3.3)**	GGCGACAGCGGTAAGATCGTG	GGTAGTAGTTGAGCACGTAGGCGA
**KCND2 (K_V4.2)**	CTTCGGCTAGCAAGTTCACC	CAGACAAACGAAGGGCACAA
**KCNH1 (K_V10.1)**	GGTAGGTCCTGTCCCTGTCA	TGCTAAATGGGCTCAGCTTT
**KCNJ1 (K_{IR}1.1)**	GTGCCAAGACCATTACGTTC	TAGCCACTCGGATTAGGAGG
**KCNJ2 (K_{IR}2.1, LQT7)**	TCAGAAGAAGACGGTATGAAGTTGG	CAGGCAGAAGATAACCAGCATCC
**KCNJ5 (K_{IR}3.4/GIRK4)**	CACCTGGCTGTTCTTCGG	TGGAGATGACTGCGTTGTT
**KCNJ9 (K_{IR}3.3/GIRK3)**	GCTTCGACACGGGAGACG	TCTCGTGAAAGCTGGCATAG
**KCNJ12 (K_{IR}2.2)**	CCCTACAGCATCGTGTCATCG	TGTTGGCGAACTCAATGTTGC
**KCNJ13 (K_{IR}7.1)**	CACTTCAAATGGATGGCGCT	CCACAAAAGCACCAGAGCAC
**KCNJ14 (K_{IR}2.4)**	CTGGTGGTCATTCTCGAGGG	AGAGCCGGGGAAACTAGACT
**KCNJ15 (K_{IR}4.2)**	CCTCATGCCATCTTCCTGTT	TTCTGCTTGGTGATGACTGC
**KCNK1 (K_{2P}1.1/TWIK1)**	AGCCTCTTCCTTGCACCCTGA	CCAACTGTCCTTCTCCTCGATC
**KCNK3 (K_{2P}3.1/TASK1)**	CGGCAAGGTGTTCTGCATG	CAAGGTGTTGATGCGCTCG
**KCNMA1 (K_{Ca}1.1/BK_{Ca} /SLO1)**	CCTGCAGCGAAGTATCATCA	ACTCGAAGTGAAGCTGCCAT
**KCNN2 (K_{Ca}2.2/SK2)**	ACACTTTGGTGGACTTGGCA	AGGTTGGTGGTGCTGTGGAA
**KCNN3 (K_{Ca}2.3/SK3)**	TGGACACTCAGCTCACCAAG	GTTCCATCTTGACGCTCCTC
**KCNN4 (K_{Ca}3.1/IK1/ SK4)**	CTGCTGCGTCTCTACCTGG	AGGGTGCGTGTTCATGTAAAG
**KCNQ2 (K_V7.2)**	GGAAACCGTTCTGTGTGATTGAC	TCCGCAGAATCTGCAGGAA
**KCNQ3 (K_V7.3)**	GCATCGGGCTCCTGGC	ACCCCAGGACAATCAGGAAC
**KCTD5 (KCTD5)**	TCCAAGTGGGTCCGACTCAA	GGTCTCTGTCGATTAAATAGGCG
**GAPDH (GAPDH)**	TCACCACCATGGAGAAGGCT	GCCATCCACAGTCTTCTGGG

Supplementary Table 1. Oligonucleotide primers used in the study for amplification of gene specific DNA in RT-qPCR analysis.

Inhibitor	Target protein (gene)	Source
**NS8593**	K_{Ca}2.1 (KCNN1), 2.2 (KCNN2), 2.3 (KCNN3)	Sigma, 492031
**NS6180**	K_{Ca}3.1 (KCNN4)	Stratech, B5740
**TRAM34**	K_{Ca}3.1 (KCNN4)	Sigma, T6700
**Clotrimazole**	K_{Ca}3.1 (KCNN4), BK_{Ca} (KCNMA1), TRPM2	Sigma, C6019
**VU590 dihydrochloride**	K_{IR}1.1 (KCNJ1) and K_{IR}7.1 (KCNJ13)	Alomone labs, V-130
**ML133**	K_{IR}2.1 (KCNJ2)	Alomone labs, M-275
**ML252**	K_V7.2 (KCNQ2)	Sigma, SML0723

Supplementary Table 2. Details of the source and known targets of the small molecule inhibitors used against K^+ channels
